# RadShap: An Explanation Tool for Highlighting the Contributions of Multiple Regions of Interest to the Prediction of Radiomic Models

**DOI:** 10.2967/jnumed.124.267434

**Published:** 2024-08

**Authors:** Nicolas Captier, Fanny Orlhac, Narinée Hovhannisyan-Baghdasarian, Marie Luporsi, Nicolas Girard, Irène Buvat

**Affiliations:** 1 Laboratoire d’Imagerie Translationnelle en Oncologie, Institut Curie, INSERM U1288, PSL Research University, Orsay, France;; 2 Department of Nuclear Medicine, Institut Curie, Paris, France; and; 3 Institut du Thorax Curie-Montsouris, Institut Curie, Paris, France

**Keywords:** artificial intelligence, explainability, radiomics, Shapley values, python package

## Abstract

Explaining the decisions made by a radiomic model is of significant interest, as it can provide valuable insights into the information learned by complex models and foster trust in well-performing ones, thereby facilitating their clinical adoption. Promising radiomic approaches that aggregate information from multiple regions within an image currently lack suitable explanation tools that could identify the regions that most significantly influence their decisions. Here we present a model- and modality-agnostic tool (RadShap, https://github.com/ncaptier/radshap), based on Shapley values, that explains the predictions of multiregion radiomic models by highlighting the contribution of each individual region. **Methods:** The explanation tool leverages Shapley values to distribute the aggregative radiomic model’s output among all the regions of interest of an image, highlighting their individual contribution. RadShap was validated using a retrospective cohort of 130 patients with advanced non–small cell lung cancer undergoing first-line immunotherapy. Their baseline PET scans were used to build 1,000 synthetic tasks to evaluate the degree of alignment between the tool’s explanations and our data generation process. RadShap’s potential was then illustrated through 2 real case studies by aggregating information from all segmented tumors: the prediction of the progression-free survival of the non–small cell lung cancer patients and the classification of the histologic tumor subtype. **Results:** RadShap demonstrated strong alignment with the ground truth, with a median frequency of 94% for consistently explained predictions in the synthetic tasks. In both real-case studies, the aggregative models yielded superior performance to the single-lesion models (average [±SD] time-dependent area under the receiver operating characteristic curve was 0.66 ± 0.02 for the aggregative survival model vs. 0.55 ± 0.04 for the primary tumor survival model). The tool’s explanations provided relevant insights into the behavior of the aggregative models, highlighting that for the classification of the histologic subtype, the aggregative model used information beyond the biopsy site to correctly classify patients who were initially misclassified by a model focusing only on the biopsied tumor. **Conclusion:** RadShap aligned with ground truth explanations and provided valuable insights into radiomic models’ behaviors. It is implemented as a user-friendly Python package with documentation and tutorials, facilitating its smooth integration into radiomic pipelines.

## Visual Abstract

**Figure:**
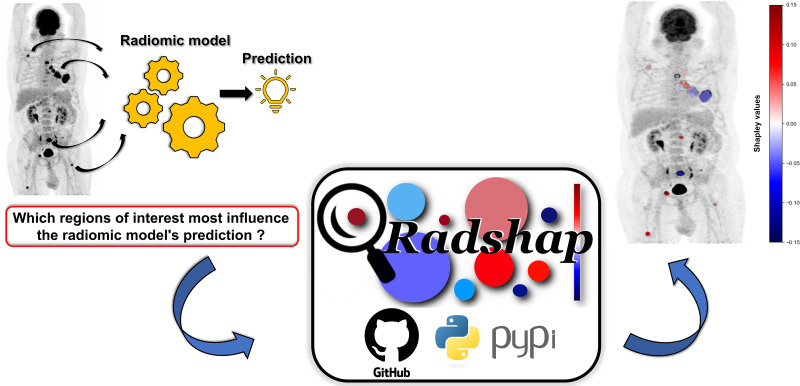

Radiomics has gained significant popularity in precision medicine, using both deep features and engineered ones that characterize shape, intensity, or texture (1). Although radiomics initially focused on a single region of interest (ROI), such as the primary tumor, more and more models now aggregate information from multiple ROIs in the same image. For instance, many radiomic approaches extract information from multiple tumors and regions within healthy organs to effectively predict cancer patients’ outcomes (2–4). The motivation is to better leverage the image content and, hopefully, improve predictive performance.

These aggregative approaches raise an interesting question: can we identify the ROIs within each image that most influence the model’s prediction? Indeed, understanding which ROIs drive the model’s decision may not only enhance the model explainability but also provide valuable medical insights. However, the tools commonly used to explain predictive models (5) may not be well suited to address this question. They usually assign a global importance to aggregated features used by the model, without straightforward identification of the role of individual ROIs.

The Shapley value, a concept originally designed to fairly distribute the overall gain of a cooperative game among its players, has recently proven successful to explain the outputs of machine learning models (6*,*^7^). It offers a promising approach to answer our question. Considering that the different ROIs of an image (i.e., the players) collaborate to obtain the model’s prediction (i.e., the overall gain), the Shapley value assigns a score to each ROI related to its contribution to the prediction.

Here we introduce an original explanation tool, named RadShap (https://github.com/ncaptier/radshap), that leverages Shapley values to highlight the influence of every ROI included into a radiomic model. We first evaluate the tool on a synthetic task for which the ground truth is known. We then illustrate its relevance through its application to radiomic models trained to address histologic classification and survival prediction tasks.

## MATERIALS AND METHODS

### Dataset

A retrospective cohort consisting of 130 individuals diagnosed with advanced non–small cell lung cancer (NSCLC) was used (Supplemental Table 1; supplemental materials are available at http://jnm.snmjournals.org) (8–10). Each patient received anti–programmed-death 1 and anti–programmed-death ligand 1 immunotherapy, specifically pembrolizumab, combined with chemotherapy, as their first-line treatment. Response to immunotherapy was assessed through progression-free survival (PFS). Clinical data and a baseline [^18^F]FDG PET scan were collected for each patient in compliance with the General Data Protection Regulation. The study was approved by the institutional review board of Institut Curie (DATA200053), and written informed consent from all patients was obtained through institutional processes.

For each PET scan, intensities were converted to SUVs and all tumor foci were delineated by an experienced nuclear medicine physician (12 y of experience) using LIFEx software version 7.3 (www.lifexsoft.org) (11). Subsequently, all images were resampled to a fixed 2 × 2 × 2 mm^3^ voxel size, and a fixed threshold of 2.5 SUV units was applied to refine the segmented tumor regions.

For all the segmented lesions, we used Image Biomarker Standardization Initiative–compliant PyRadiomics version 3.0.1 (12*,*^13^) to compute 4 shape radiomic features (sphericity, elongation, flatness, and voxel volume) and 6 first-order radiomic features (SUV_max_, SUV_mean_, skewness, kurtosis, entropy, and quantile dispersion).

### Computation of Shapley Values for Local Explanations

The RadShap explanation tool provides a local explanation for each prediction made by a radiomic model. This is achieved by calculating the Shapley values (6) of the aggregated ROIs used as input, assigning an importance score to each ROI based on its contribution to the final prediction. In short, the Shapley value of an ROI quantifies the impact of including this ROI in the model on the model’s prediction. It corresponds to the average change in prediction observed when the ROI is added to any combination of other ROIs in the predictive model’s input. The Shapley values distribute the model’s output among the various ROIs, as their sum corresponds to the model’s overall prediction.

**FIGURE 1.**
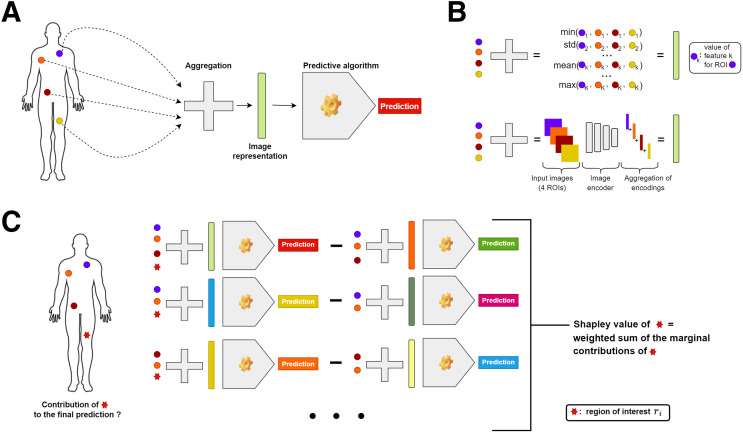
(A) Schematic representation of generic aggregative radiomic model. (B) Examples of aggregation function with engineered radiomic features (top) or deep radiomic features (bottom). (C) Schematic representation of explanation tool.

For an input image *I* characterized by *K* ROIs ($r_{1},r_{2}\ldots ,\;r_{K})$, a trained predictive model *f*, and an aggregation function g (Figs. 1A and 1B), the Shapley value of region $r_{i}$ is calculated by averaging its marginal contribution across all possible subsets of ROIs that exclude $r_{i}$. The marginal contribution of $r_{i}$ to the subset of ROIs *S* is the difference observed in the model’s prediction when $r_{i}$ is included in subset *S* (Fig. 1C).

$$\begin{array}{c} {\text{Δ}}_{f\circ g}\left(r_{i},\;S\right)=f\left(g\left(S\cup^{\text{​}}\{r_{i}\}\right)\right)-f\left(g\left(S\right)\right)\; \end{array}$$ (Eq. 1)

A detailed mathematic formulation is presented in Supplemental Section A, along with the description of a computational approximation scheme used to speed the estimation.

RadShap is both model- and modality-agnostic and, thus, applicable to any trained radiomic model that uses aggregated information from multiple ROIs as input. In the standard setting, the ROIs are assumed to be optional, meaning that aggregating any subset of ROIs will still produce a valid input for the predictive model. For instance, if the predictive model takes as input the average volume computed across all lesions of a metastatic patient, each lesion is optional. Indeed, removing a lesion will result only in the average volume’s being computed across the remaining lesions, still yielding a valid input for the model. However, if the model also considers the SUV_max_ in an ROI delineated in a healthy spleen region (i.e., SUV_max(spleen)_), this ROI is not optional since removing it will result in a missing value for the SUV_max(spleen)_ feature and therefore an invalid input for the predictive model. Our explanation tool includes a solution to address the scenario in which certain ROIs are not optional. Following a strategy used in the Shapley additive explanation (SHAP) method (7), the missing values resulting from the removal of nonoptional ROIs are replaced by all possible values observed in a background dataset (i.e., set of data samples representative of the general data distribution that the model is expected to encounter, typically the training set). The final prediction is then obtained by averaging the predictions made with every possible background value (Supplemental Sections B and C).

### Building a Synthetic Task

**FIGURE 2.**
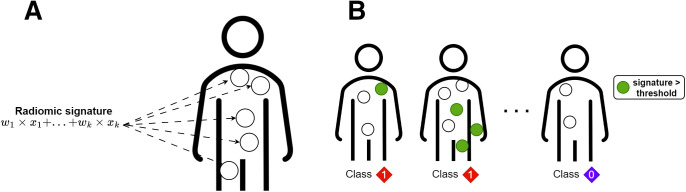
(A) Computation of synthetic radiomic signature for every lesion of each patient. (B) Each patient was assigned binary label based on presence of high-scored lesions, meaning lesions with radiomic signature above specified threshold.

To validate the explanation tool, we first built a synthetic radiomic signature as a linear combination of a random subset of the 10 radiomic features listed above and computed the signature value for every lesion of each NSCLC patient (Supplemental Section D). A binary label was then assigned to each patient: 1 if the patient had at least 1 lesion with the radiomic signature value above a predetermined threshold and 0 otherwise. The threshold was selected to ensure that the 2 classes were reasonably balanced, with the minority class frequency exceeding 30%. To achieve this, the maximum signature value was calculated for each patient, and the q^th^ percentile (with q ranging from 30% to 70%) was randomly selected. This ensured that over q% of the patients had at least 1 lesion with a signature value exceeding this threshold, resulting in their label being set to 1. To enhance the robustness of our results, we repeated this experiment 1,000 times, with a different radiomic signature and a different threshold for each iteration (Fig. 2).

We then addressed this binary classification task using a logistic regression model with ridge regularization (i.e., scikit-learn implementation with default parameters and balanced class weights). The model’s input consisted of the 10 radiomic features measured for all the patient’s lesions. Specifically, these features were aggregated into a 20-feature vector using min and max functions, which involved computing the minimum and maximum of each of the 10 features across all lesions and concatenating these 20 values in a single vector. Importantly, no information related to the radiomic signature or label generation was provided to the model. Training and test were performed with a stratified 5-fold cross-validation scheme. To collect the Shapley values associated with each patient’s lesions, our tool was applied to every patient within the test set of each fold using the model trained on the corresponding training set.

### Validation of the Tool Using a Synthetic Task

When the model accurately predicted the binary labels, we assumed that the model used information related to the presence of lesions with a high radiomic signature to make its decision. Thus, we evaluated the consistency of the tool’s explanations by measuring the frequency with which the model correctly predicted a “1” label and the Shapley values identified a lesion with a high radiomic signature as the most influential factor affecting the model’s decision. We also calculated the number of true-positive cases for which all lesions with a high radiomic signature value were assigned a higher Shapley value than any other lesion with a low signature value.

### Survival Task: Explaining the Prediction of PFS Under Immunotherapy

The first case study addressed the prediction of PFS, comparing a standard approach involving radiomic features extracted from the segmented primary tumor (called primary model hereafter) with an approach using all segmented lesions (called aggregative model hereafter). Specifically, within the aggregative model, we used as input 2 multilesion features: the total metabolic tumor volume and the standardized D_max_—defined as the maximum distance between 2 lesions normalized by the body surface area—a metric previously associated with outcomes in lymphoma patients (14). In total, we identified 115 NSCLC patients for whom both the primary tumor was visible on the PET scan and PFS data were available.

In the primary model, we included the 10 radiomic features extracted by PyRadiomics and listed in the Dataset section above. We then applied 2 successive feature selection preprocessing steps each time the primary model was trained. Initially, a backward elimination process based on the variance inflation factor was used to remove collinear features. Subsequently, another backward elimination step based on the Akaike information criterion was applied to discard unnecessary features for prediction. Both the primary and the aggregative models were trained using a Cox proportional-hazards algorithm (default settings of Lifelines Python package).

### Classification Task: Explaining the Prediction of Lung Cancer Histologic Subtype

The second case study addressed the prediction of the NSCLC histologic subtype (adenocarcinoma vs. other subtypes). We compared a standard approach that used features extracted only from the biopsied lesion (called biopsy model hereafter) with an approach that aggregated features extracted from all segmented lesions (called aggregative model hereafter). We excluded patients for whom the biopsied tumor was not present in the PET scan or the localization of the biopsy site was not available, resulting in a final dataset of 117 patients (87 adenocarcinomas and 30 other subtypes). For 88 patients, the biopsy was performed on their primary lung tumor, whereas for the remaining 29 patients, the biopsy was performed on another lesion.

A logistic regression model with elastic net regularization (i.e., scikit-learn implementation with balanced class weights, C = 0.1, and l1_ratio = 0.5) was used for both biopsy and aggregative models. For the aggregative model, several combinations of simple functions were tested to aggregate the feature values extracted from all the lesions of a patient (min, max, mean, and SD).

### Statistical Analysis

For the 1,000 synthetic tasks, the significance of the tool was assessed using a right-tailed test associated with the null hypothesis: the explanations were generated with a random ranking of lesions, and therefore, the number of consistent explanations followed a Poisson binomial distribution. We report the number of tasks for which this hypothesis was rejected after controlling the false discovery rate with the Benjamini–Yekutieli procedure.

For the PFS prediction and histologic classification studies, the models were trained and tested using a 5-fold cross-validation scheme repeated 100 times. The folds were stratified on the basis of censorship rate for the survival task (i.e., PFS prediction) and class proportion for the classification task (i.e., histologic classification). The performance of the binary classifiers was evaluated using 4 metrics: the area under the receiver operating characteristic curve (AUC), balanced accuracy, sensitivity, and specificity (0.5 threshold). For Cox models, evaluation was based on 2 metrics: Uno’s concordance index (C-index) and the average time-dependent AUC (tAUC) over the observed time range. These metrics were averaged over 100 repetitions, and their SD was calculated to measure the variability resulting from the random partition of the data into 5 folds. The AUCs and C-indices were compared with 1-sided paired permutation tests (15), adjusted for multiple testing. Finally, an additional cross-validation scheme was used to collect the Shapley values associated with the lesions of each patient for the aggregative models.

## RESULTS

### Synthetic Tasks

**FIGURE 3.**
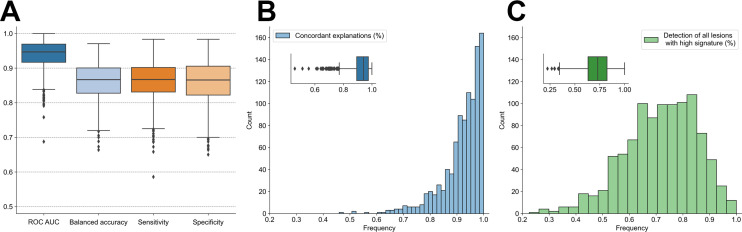
(A) Box plots of performance of logistic regression model trained and tested with stratified 5-fold cross-validation scheme across 1,000 synthetic tasks. (B) Distribution across 1,000 tasks of frequency of true-positive cases for which RadShap provided explanation aligned with synthetic data generation process, ranking lesion with high radiomic signature value as most impactful lesion. (C) Distribution across 1,000 tasks of frequency of true-positive cases for which RadShap ranked all lesions with high signature value above any lesion with low signature value.

The logistic regression model trained using aggregated radiomic features demonstrated high classification performance: for half the 1,000 synthetic tasks, it yielded a cross-validation AUC greater than or equal to 0.95 (Fig. 3A). Furthermore, both the cross-validation sensitivity and specificity median values were 87% (Supplemental Fig. 1). This demonstrates the model’s ability to learn discriminative information for accurate patient classification in this synthetic setting.

In half the experiments, for more than 94% of the true-positive cases the explanation tool assigned the highest importance to a lesion with a high radiomic signature value (Fig. 3B; Supplemental Fig. 2A). For 997 experiments of 1,000, the number of these consistently explained cases was significant against a random ranking of lesions (false discovery rate controlled at level 0.001; Supplemental Fig. 3). In 50% of the experiments, for more than 73% of the true-positive cases the explanation tool ranked all the lesions with a high signature value above any lesion with a low signature value (Fig. 3C; Supplemental Fig. 2B).

### Survival Task

**FIGURE 4.**
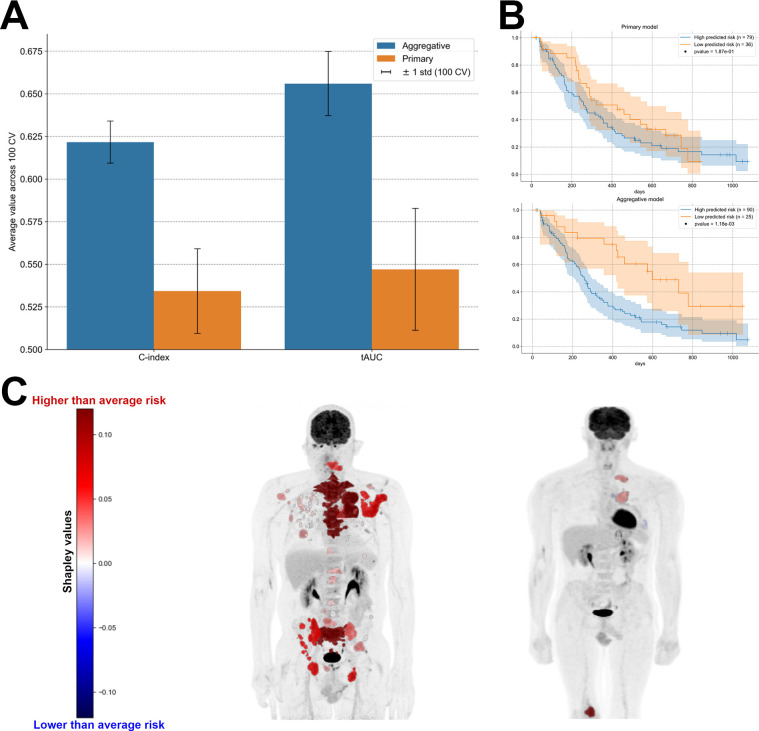
(A) Comparison of performance between primary and aggregative survival models for predicting PFS. (B) Kaplan–Meier survival curves for group of patients with high-risk predictions and group of patients with low-risk predictions for both primary (top) and aggregative (bottom) models. Predictions were collected from test sets of cross-validation scheme, and thresholds to define high- and low-risk groups were selected to maximize log-rank statistic. (C) Shapley values for explaining high-risk predictions made by aggregative model for 2 patients with high predicted risk, displayed on maximum-intensity projection of each [^18^F]FDG PET scan.

The aggregative model outperformed the primary model for both C-index and tAUC metrics (mean tAUC primary [±SD], 0.55 ± 0.04; mean tAUC aggregative, 0.66 ± 0.02) (Fig. 4A). The difference in C-index between the primary and aggregative models was statistically significant in 60 of 100 repetitions of the cross-validation scheme, after correction of *P* values for multiple testing using the Benjamini–Hochberg method (false discovery rate controlled at level 0.05) (Supplemental Fig. 4). Additionally, Kaplan–Meier analysis demonstrated that predictions of the aggregative model, obtained from the test sets of a cross-validation scheme, resulted in better risk stratification than did predictions of the primary model, obtained from the same cross-validation scheme, as measured by the log-rank test *P* values (Fig. 4B).

The RadShap tool offered explanations consistent with our understanding of the aggregative model, which combines total metabolic tumor volume and standardized D_max_. Specifically, for each patient in a test set of the cross-validation scheme, RadShap ranked lesions on the basis of their influence on the aggregative model’s prediction, correctly identifying large and distant lesions as the most impactful for high-risk predictions (Fig. 4C).

### Classification Task

**FIGURE 5.**
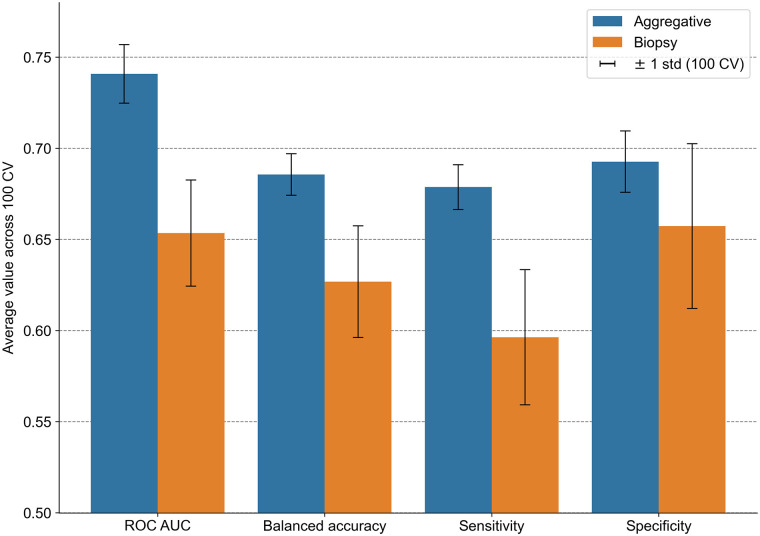
Comparison of classification performance between biopsy and aggregative classification models for prediction of lung cancer subtype. CV = cross-validation; ROC AUC = area under receiver operating characteristic curve.

The maximum values of the 10 radiomic features across all segmented lesions consistently yielded the highest average classification performance for the 100 cross-validation schemes. This approach outperformed the model trained on features extracted from only the biopsied tumor for all figures of merit (Fig. 5; Supplemental Fig. 5). Although the increase in AUC was not significant with paired permutation tests (Supplemental Fig. 6), the observed improvements still motivate the use of our explanation tool to gain insights into the complementary information leveraged by the aggregative model.

**FIGURE 6.**
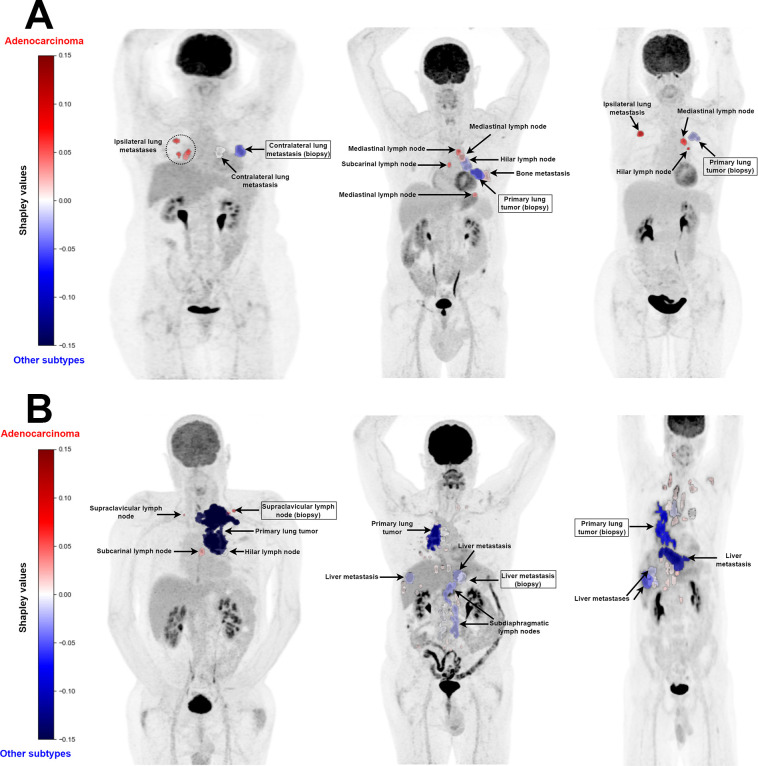
Shapley values for explaining correct prediction made by aggregative model, displayed on maximum-intensity projection of each [^18^F]FDG PET scan. (A) Shapley values for 3 patients diagnosed with adenocarcinoma who were correctly classified by aggregative model and misclassified by biopsy model. (B) Shapley values for 3 patients diagnosed with nonadenocarcinoma subtype who were correctly classified by aggregative model and misclassified by biopsy model.

In the test sets, predictions differed between the biopsy and the aggregative model for 28 patients of 117. Among those, the aggregative model correctly classified 18 of 28 patients (64%). For patients diagnosed with adenocarcinoma (15/18), the explanation tool consistently highlighted that the biopsied tumor influenced the model’s prediction toward a nonadenocarcinoma subtype (or had no influence in 1 case), whereas at least 1 metastasis directed it toward an adenocarcinoma. This observation confirmed that the sensitivity of the aggregative model was increased by the integration of information beyond the biopsied tumor. Our tool unveiled such information, prompting further exploration to determine whether the highlighted metastatic patterns are indeed related to adenocarcinoma (Fig. 6). Lastly, for the 3 patients for whom the aggregative model, unlike the biopsy model, correctly identified a nonadenocarcinoma subtype, the biopsied lesion influenced the model’s prediction toward the correct subtype in 2 cases. This observation suggests that the aggregative model did not retain the same information as the primary model, leading to distinct considerations for certain biopsied tumors.

## DISCUSSION

Many radiomic studies now use explanation tools to gain insight into the behavior of their predictive models (16). However, new approaches that involve combining multiple regions within an image to comprehensively characterize patient phenotypes and thereby build powerful predictors still lack well-suited explanation tools. Tools capable of highlighting the most impactful regions within an image hold great interest, as they could help in deciphering the information learned by predictive models, detecting potential biases (e.g., a region with no clear medical relevance is consistently highlighted across patients), or providing valuable medical insights (e.g., within all the metastases of a patient, a subset is identified as strongly associated with the prediction of the patient’s outcome). Some studies proposed strategies, such as using attention weights (17), to assess the contribution of individual regions to a prediction, but these methods were closely tied to the developed predictive model. In this study, we introduced a novel model- and modality-agnostic tool to explain the outputs of multiregion radiomic models by highlighting the impact of each individual region on the model prediction.

The RadShap explanation tool was first validated with 1,000 synthetic classification tasks. In each task, a binary label was assigned to each patient within a cohort of 130 metastatic NSCLC cases, based on the presence of lesions with a high value of a randomly defined radiomic signature. Subsequently, a model, blinded to the data generation process, was trained to predict the binary label from radiomic features aggregated at the patient level. As expected, across the 1,000 tasks, most of the models were able to accurately classify patients and learn the underlying label generation process. RadShap successfully provided explanations well aligned with the ground truth, highlighting the pivotal role of lesions with a high signature value and, consequently, retrieving the label generation process learned by the models.

We then applied RadShap to the prediction of PFS for metastatic patients undergoing first-line immunotherapy and the classification of their histologic subtype, using their baseline [^18^F]FDG PET scan. Cross-validation experiments demonstrated an increase in performance for both survival and classification tasks when aggregating information from all lesions of each patient, compared with radiomic models relying solely on a single lesion—either the primary tumor for the survival task or the biopsied lesion for the classification task. For the survival task, a model combining total metabolic tumor volume and standardized D_max_ significantly outperformed a model based on the primary tumor only, thus confirming the promising predictive and prognostic value of these 2 aggregated features for metastatic NSCLC (18*,*^19^). The RadShap explanations offered valuable insights into understanding the behavior of the aggregative models and their differences with the single-lesion models. Specifically, for the classification task, it highlighted that for most patients correctly classified by the aggregative model and misclassified by the biopsy model, information from the biopsied tumor influenced the aggregative model toward an incorrect prediction, whereas information from other lesions guided it toward the correct prediction.

We implemented RadShap in a user-friendly Python package and made it available for the radiomic community. It is applicable to any radiomic models that aggregate information from multiple regions as input, with the most appropriate setting being when all the regions are optional with the aggregation function (as described in the Materials and Methods section). Furthermore, RadShap is a fast-running tool, as it applies to an already trained radiomic model and uses only its prediction function; there is no need to retrain the model. RadShap explanations are grounded in the robust theoretic background of the Shapley values. In contrast to several explanation methods, such as SHAP (7), in the standard setting (i.e., when the ROIs are optional), there is no need to approximate the effect of removing an ROI from the predictive model to compute its marginal contribution.

The use of RadShap requires some Python coding skills, especially for defining custom aggregation functions. However, Python is a widely used programming language within the radiomic community for developing machine learning models. Additionally, like other explanation tools, RadShap offers no guarantee regarding the validity of the information that the model learned. Its application should be coupled with rigorous validation experiments to evaluate the robustness and generalization ability of the model. Finally, making sense of the impactful regions highlighted by RadShap is not always straightforward. Leveraging medical expertise and conducting additional experiments can aid in formulating relevant hypotheses based on the RadShap explanations.

Our study had limitations. First, we worked with a limited number of samples in the real case applications. Therefore, the performance scores should be interpreted cautiously, although they were not the primary focus of this study. Additionally, the aggregative model used for the survival task could have been explained with human intuition alone. Nevertheless, this task demonstrated that RadShap’s explanations aligned with human understanding in a real-world context and highlighted the potential benefits of multiregion radiomic models, thereby supporting the relevance of RadShap. Finally, whereas we provided supportive evidence for the utility of the tool in elucidating complex aggregated strategies, we did not explore its full potential across a wide range of real-world scenarios and models. We believe that such a comprehensive evaluation will naturally emerge as the community starts experimenting with it. This is why we have made substantial efforts to ensure that our tool is easily and freely accessible through a user-friendly Python package.

## CONCLUSION

We developed and validated a tool, implemented as the Python package RadShap, to offer local explanations for decisions of radiomic models involving several regions of interest. It complements the existing explanation strategies, focusing on multiregion approaches, to improve the understanding of radiomic models. These efforts toward explainable radiomics might both facilitate the adoption of well-performing models and provide relevant medical insights.

## DISCLOSURE

This work was supported by Fondation ARC (TIPIT project SIGNIT202001322) and the French national agency ANR as part of the “Investissements d’avenir” program, reference ANR-19-P3IA-0001 (PRAIRIE 3IA Institute). No other potential conflict of interest relevant to this article was reported.

## Notes

KEY POINTS **QUESTION:** Can we identify the ROIs within an image that most influence a radiomic model’s prediction? **PERTINENT FINDINGS:** Our explanation tool demonstrated a strong alignment with the ground truth for 1,000 synthetic tasks. It also provided relevant insights to understand multilesion radiomic models’ behavior, highlighting the most impactful lesions for the classification of the NSCLC subtype or the prediction of PFS. **IMPLICATIONS FOR PATIENT CARE:** RadShap may improve the understanding of well-performing radiomic models, thus fostering their adoption in clinical practice.
